# Uncertainty displays in pandemic dashboards: a review of COVID-19 use cases

**DOI:** 10.3389/fdgth.2026.1869108

**Published:** 2026-07-30

**Authors:** Tselot Tessema, Marta Shahezian, Holland Vasquez, Laura Rosella, Birsen Donmez

**Affiliations:** 1 Human Factors and Applied Statistics Laboratory, Department of Mechanical and Industrial Engineering, University of Toronto, Toronto, Ontario, Canada; 2 Population Health Analytics Laboratory, Dalla Lana School of Public Health, University of Toronto, Toronto, Ontario, Canada

**Keywords:** COVID-19, data dashboards, data visualization, decision-making, public health, risk communication, uncertainty, uncertainty visualization

## Abstract

Pandemic dashboards are a key resource that public health professionals and the public use to access timely, dynamic, and multivariate public health data. Epidemiological data inherently carry uncertainty due to factors related to location, time, and the movement of people and the spread of disease. Uncertainty information has been neglected in dashboard design due to some technical challenges, including the complexity of communication, and to avoid potential misinterpretation. However, including uncertainty information may improve transparency and dashboard actionability in public health. This study scanned Canadian COVID-19 dashboards to assess how uncertainty was communicated during the pandemic. A total of 69 dashboards were analyzed in 2023. The findings show that most COVID-19 dashboards did not meet best practices for improving actionability and usability, nor did they include uncertainty data. Only 1 of 69 dashboards included a visualization of uncertainty, highlighting a substantial gap between established uncertainty visualization research and real-world public health dashboard design. Additionally, most dashboards lacked a clear target user and did not provide sufficient resources to support public use. Future research can investigate how dashboard users engage in public health decision-making and change their health behaviours using uncertainty information.

## 1 Introduction

During pandemics, dashboards have emerged as a pivotal tool for disseminating information to a wide audience, including public health professionals, government and health system decision-makers, and the public. Dashboards have been valuable tools, serving as “graphic user interfaces which comprise a combination of information and geographical visualization methods for creating metrics, benchmarks, and indicators to assist in monitoring and decision-making” (1). COVID-19 pandemic dashboards, many of which are accessible online, provided real-time data on various public health indicators, including case counts, deaths, and recoveries, both globally and in specific regions. For instance, the Johns Hopkins University COVID-19 dashboard displayed real-time data on global and local cases, deaths, and recoveries, featuring an interactive map that allowed users to click on specific regions for detailed statistics, along with trend graphs and charts for comprehensive data analysis. Similarly, government and health organization dashboards at national and local levels offered tailored information relevant to specific demographics. These dashboards helped monitor the pandemic and understand trends, predict hotspots, and assess the effectiveness of interventions like lockdowns and vaccination campaigns and played a critical role in enhancing transparency and facilitating informed decision-making. As such, their use underscores the importance of data visualizations in managing public health crises.

It is important for users of pandemic dashboards to understand that all epidemiological data inherently carry some degree of uncertainty due to factors like reporting delays, testing availability, and differing data collection methods across regions to accurately interpret the data and any model predictions (2, 3). Acknowledging and clearly communicating uncertainty in data and models is essential, especially in public health contexts, as it fosters public trust by setting realistic expectations and may prevent the erosion of confidence when predictions and outcomes differ (4–6). Additionally, understanding uncertainty aids decision-makers in effectively allocating resources, such as vaccines and medical supplies, by planning for various scenarios, from best-case to worst-case, and allows for more adaptable and robust policymaking, ensuring strategies can flexibly respond to a range of potential future situations (3, 7, 8). The increasing adoption of complex algorithms, such as machine learning (ML) and artificial intelligence (AI), for predictions further underscores this need (5, 9, 10).

A framework for understanding uncertainty in health contexts is provided by Han et al. (3), who distinguish between probability, ambiguity, and complexity as three fundamental sources of uncertainty. In this framework, probability refers to the inherent randomness of natural events (aleatory uncertainty), ambiguity emerges from incomplete or unreliable information (epistemic uncertainty), and complexity refers to difficulties in understanding due to the structure or volume of information. This taxonomy captures the underlying causes of uncertainty and offers a more actionable lens for communication and design. For example, uncertainty resulting from reporting delays or inconsistent testing practices reflects ambiguity and may require different communication strategies than probabilistic variation in case counts. Integrating this perspective into dashboard design practice can help guide the selection of uncertainty displays that are better aligned with the nature of the public health data and needs of decision-makers.

There are other characterizations uncertainty that can be considered in public health decision-making, namely statistical uncertainty and geospatial uncertainty (11). Statistical uncertainty describes the difference between a prediction estimate and its true value. Geospatial uncertainty describes this difference within the context of its geographic data (i.e., location, time, and movement). Sources of geospatial uncertainty include delays in the onset of disease symptoms, delays in reporting, and delays in diagnosis, which affect public health prediction estimates.

A growing body of research suggests how cognitive processing of uncertainty visualizations can affect user interpretations and decision-making. Padilla et al. (11) describe a dual-process system of decision-making (12). The first process (i.e., Type 1) is fast and intuitive, where individuals apply information about graphical data interpretation stored in their long-term memory using a heuristic-based approach. The second process (i.e., Type 2) is deliberate and effortful, where individuals either apply information held temporarily in their working memory (e.g., from a legend or other visualization notes) or must analyze information in their long-term memory to interpret the visualization. The design of the uncertainty visualization can influence which cognitive process is engaged. For example, displays requiring statistical interpretation, such as confidence intervals, tend to impose higher cognitive demands (Type 2) and are more prone to misinterpretation. By contrast, visualizations that map uncertainty onto frequencies or concrete outcomes can better support intuitive reasoning (Type 1). More recent syntheses of the uncertainty visualization literature further emphasize that different visual encodings (e.g., probabilistic, ensemble, frequency displays) can cause different judgments, levels of trust, and decision quality among users (13). These findings underscore the importance of designing uncertainty representations that align with human perceptual and cognitive capabilities.

While dashboards like the Johns Hopkins University COVID-19 dashboard provided invaluable real-time data on the pandemic, they did not include textual descriptions, numerical estimates, or visualizations of uncertainty, such as confidence intervals or error margins. Confidence intervals and standard error bars are common methods to visualize uncertainty. Confidence intervals provide an interval expected to contain the parameter being estimated a percentage (e.g., 95%) of the time across infinite studies (14). Research shows that confidence intervals are often misinterpreted; thus, information displays containing this metric could benefit from a description of their correct interpretation. Incorporating uncertainty information into dashboard design is crucial as it enhances the reliability and transparency of the data presented, enabling users to make more informed decisions based on the range of possible outcomes (7, 15). Uncertainty information can be especially important for dashboards powered by predictive analytics and AI (5). Studies have shown that clinicians mistrust predictive models and AI when the uncertainty information is unclear or visually unappealing (16). Recent research has found that visualizing uncertainty enhances trust in AI models and reduces trust in geographic information systems (17, 18).

Uncertainty information can be incorporated into dashboards through a range of visualization techniques that differ in how they encode variability and support interpretation. A common approach is the use of statistical encodings (e.g., confidence intervals, error bars, and shaded ranges), which represent the spread or variability around a central estimate. Although widely used, these techniques require statistical literacy and are prone to misinterpretation. A second approach involves frequency-based representations (e.g., hypothetical outcome plots, quantile dotplots, and icon arrays), which translate uncertainty into repeated or discrete outcomes; these formats have been shown to better align with intuitive (Type 1) reasoning and can improve users' decision-making under uncertainty (19–21). A third category includes geospatial uncertainty displays, such as probabilistic maps, gradient surfaces, and ensemble visualizations, which illustrate how uncertainty varies across locations and scenarios. Interactive features or controls allow users to explore how changes in variables affect the data, offering a hands-on understanding of uncertainty. Lastly, standalone textual descriptions or annotations in combination with visualizations can communicate the sources and implications for decision-making using uncertainty data, making the data more accessible. Each approach reflects different assumptions about cognitive processing and users' statistical knowledge. When thoughtfully designed, visual representations of uncertainty can support accurate interpretation and decision-making.

Despite its importance, uncertainty information is often neglected in dashboard design potentially due to several practical challenges. One significant factor is the complexity of communicating uncertainty in a way that is understandable to a broad audience, as it can add a layer of complexity that might confuse users. Differences in statistical knowledge amongst users and biases in human decision-making can also affect how uncertainty information is interpreted (9). Additionally, there may be limitations in the available data itself, with some datasets lacking the necessary detail to accurately represent uncertainty. Technical constraints in dashboard design can also play a role, as incorporating sophisticated visualizations of uncertainty might require more advanced and resource-intensive programming. Lastly, there are concerns about public perception; poorly communicated uncertainty may reduce confidence in the data or lead to confusion or skepticism among the public (4, 22, 23). These factors can lead to the omission of uncertainty information in dashboards, despite its significance for providing a complete understanding of the data.

An environmental scan is a process or tool used to assess the working environment around and within a health issue (24). In public health, environmental scans are often used during policy development and assessments. This method can also help researchers contextualize and focus their design research, increase recognition of prior design work, and identify gaps in current design practice.

Researchers have conducted environmental scans of COVID-19 dashboards since the onset of the global pandemic in 2019. The COVID-19 pandemic presents a unique research opportunity to gather insights about modern dashboards developed for surveillance and decision-making under uncertainty. This research scope also controls for differences in design practices that could vary according to the public health disease context. Ivankovic et al. (25) gathered health care performance intelligence researchers together to assess public, web-based COVID-19 dashboards for their actionability. Dashboards are actionable when the information displayed is usable and suits the decision-making task (26). After their assessment, the group consolidated a list of seven features found amongst the COVID-19 dashboards with the highest actionability scores, including information about the target audience, data sources, geographic filters, population filters, and visual cues. Barbazza et al. (27) conducted an environmental scan to examine the evolution of Canadian COVID-19 dashboard designs based on the actionability criteria discussed in Ivankovic et al. (25). Ansari & Martin (28) also conducted an environmental scan of publicly available dashboards, which led to the development of a usability checklist; scientific integrity, understandability, and readability were among the eleven items listed. Researchers also recommend including text-based narratives alongside quantitative information displays, audience segmentation, a range of data aggregation displays, geospatial uncertainty, and metadata in dashboards to provide users with the information required for appropriate data-driven decision-making (29). These articles highlight the need for public health dashboards with designs that enhance the tool's actionability and usability for decision-making. Despite research on effective uncertainty visualization techniques, it is unclear whether these approaches have been adopted in the design of such tools.

The primary objective of this study is to learn whether and how uncertainty displays and explanations have been incorporated into the designs of Canadian COVID-19 dashboards. The findings will help identify potential gaps in the design practices of national, provincial, and municipal dashboards and contribute to the literature on developing actionable public health dashboards. The study also expands the dashboards analyzed in Barbazza et al. (27), which reported on dashboards created until May 2020. The present investigation considers a larger sample of dashboards created from the onset of the pandemic until September 2023.

## 2 Methods

### 2.1 Study design

An environmental scan of Canadian COVID-19 dashboards was conducted. The first round of data extraction was conducted manually during a 2-month period from January 18th to March 7th, 2023. A second round of data extraction was conducted on August 30, 2023 using ChatGPT (GPT-4) to expand the number of dashboards reviewed (30). See section 2.5 Expanding the Dashboard Sample Using AI Tools for more details.

### 2.2 Dashboard sampling

We included Canadian COVID-19 dashboards based on the following criteria: (1) contains Canadian data, (2) contains public health indicators related to the COVID-19 pandemic, (3) the use of at least one data visualization, and (4) publicly available on the Internet. Dashboards that were not created in English were excluded.

The Barbazza et al. (27) study was consulted for a list of Canadian dashboards developed at the national, provincial, and municipal levels. In addition, a Google search strategy was used where the top hits from the following searches were compiled: “Canadian COVID-19 dashboards” and “[insert Canadian health region] COVID-19 dashboard”. One reviewer then cross-referenced the dashboards found from the Barbazza et al. paper and Google search strategy to remove duplicates.

### 2.3 Data extraction

The data extraction categories were adapted from previous studies that characterize highly actionable dashboards, such as Ivankovic et al. (25) and Barbazza et al. (27). We also added categories related to uncertainty information, which was inspired by the discussion of narrative explanations for communication and reporting gaps in public health data (29). Overall, we defined five categories of information for data extraction: (1) creator information (i.e., public or private author), (2) descriptive features of the dashboard (i.e., audience and purpose, types of visualizations, indicators, and technology used for development), (3) functional features (i.e., types of interactive), (4) uncertainty information (i.e., type of uncertainty, display of uncertainty, and narrative explanation), and (5) metadata (i.e., definition of indicators, forecasting model assumptions, and dashboard data sources).

We converted the data extraction criteria into survey questions in REDCap. The REDCap survey simply served as a more efficient and organized method of data collection vs. manual data entry on a spreadsheet. Once the environmental scan was complete, the data was downloaded from REDCap as a CSV file for analysis.

Four reviewers were trained for data extraction using three of the dashboards. Each reviewer completed the extraction independently, and then the reviewers reconvened to discuss the discrepancies and refine the extraction criteria. After training, the dashboards were assigned to each reviewer, with two independent reviewers extracting data from 33 of the dashboards. Discrepancies in the data extraction were resolved by a third reviewer. Any discrepancy that could not be resolved by the third reviewer was discussed by all reviewers until a consensus was reached.

Inter-rater reliability was assessed using Cohen's kappa for the subset of dashboards that were independently coded by two reviewers (*n* = 39). Kappa was calculated for each categorical variable based on initial independent ratings prior to consensus resolution. For each extraction variable, Cohen's kappa was calculated using the paired ratings from the two assigned reviewers; dashboards for which one or both reviewers left an item blank or selected “Other” were excluded from the kappa calculation for that variable. Of the 39 dashboards, 33 had pre-consensus independent ratings available and were included in the kappa calculation; for the remaining 6, only post-consensus scores were retrievable and these were therefore excluded from the inter-rater reliability analysis. The remaining dashboards (*n* = 30) were extracted using AI-assisted methods, verified through manual audit, and therefore not included in the inter-rater reliability analysis.

### 2.4 Creating a taxonomy for categorizing dashboards by functionality

In identifying dashboard purpose, we created a taxonomy to categorize dashboards by the highest order functionality. *Summary reporting* describes dashboards that contain static visualizations and do not get updated frequently. Typically, these reports are created for high-level reporting and are not consulted for day-to-day decision-making. *Monitoring and surveillance* describes dashboards that typically have filters with multiple geographic regions for comparison and are updated frequently (i.e., weekly, bi-weekly, or monthly). These geographic specifications and up-to-date information are useful for public health professionals in front-line decision-making and health promotion roles. *Forecasting* describes dashboards that utilize predictive models to estimate future data. This type of dashboard is useful for public health professionals involved in planning resource allocations and developing policy interventions.

### 2.5 Expanding the dashboard sample using AI tools

Generative AI was used to support the identification and structured extraction of dashboard information during the second phase of data collection. Specifically, ChatGPT (OpenAI, GPT-4, model version gpt-4-0613) was used through the consumer chat interface in conjunction with the “Scraper” plugin (gafo.tech) to retrieve content from dashboard webpages and the “Make a Sheet” plugin to organize extracted information into a structured tabular format. A standardized prompt was developed to guide the extraction of predefined variables aligned with the study's data collection framework (see Supplementary Material).

All AI-assisted outputs were manually reviewed and verified by multiple reviewers to ensure accuracy, completeness, and consistency with the study criteria. Any discrepancies or missing information were corrected through manual inspection of the source dashboards. The AI tools were used solely to assist with data retrieval and organization and did not perform independent analysis or interpretation of the findings.

### 2.6 Ethics

This study analyzed publicly available COVID-19 dashboards. Ethics approval was not required.

## 3 Results

**Figure 1:**
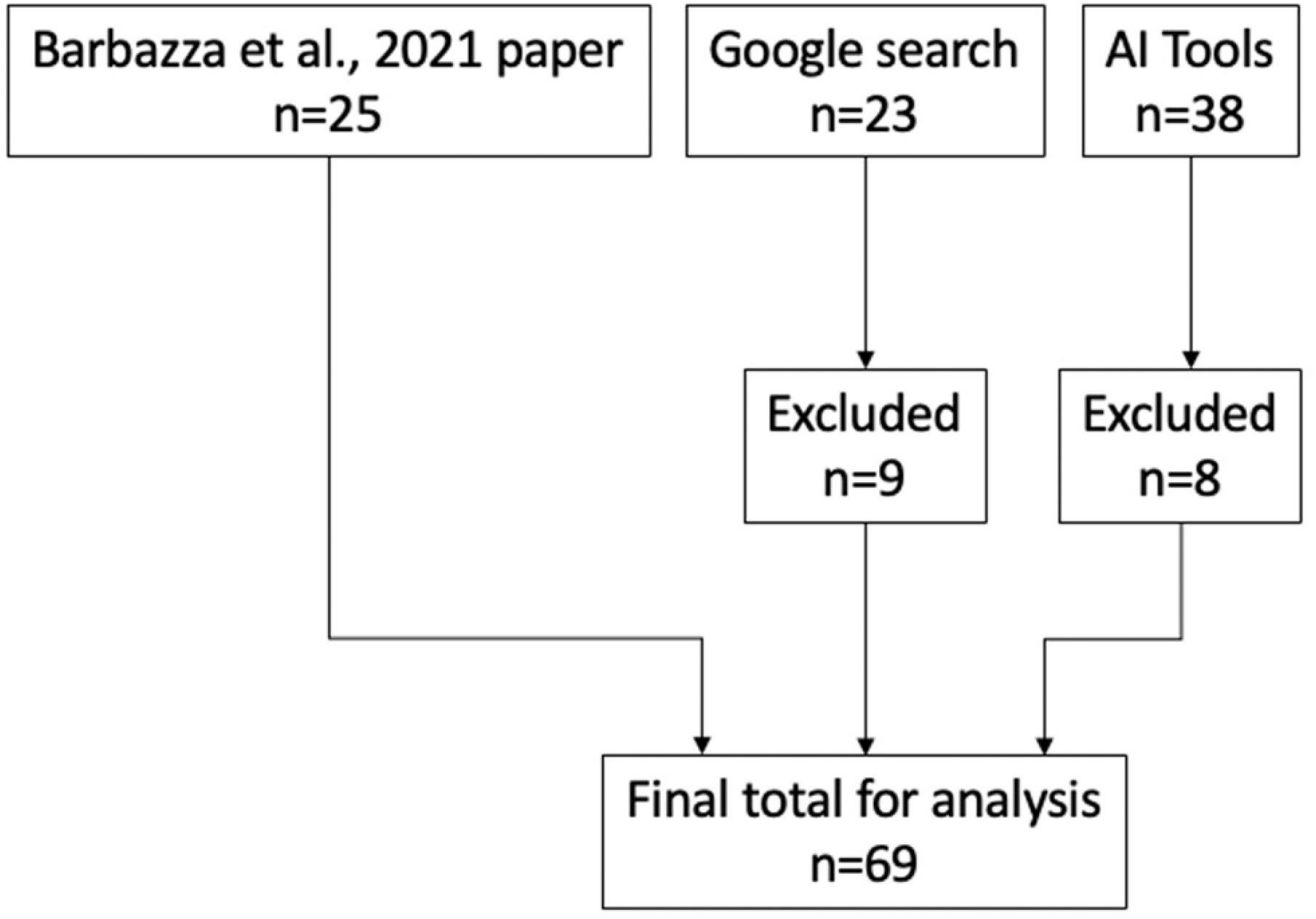
Flow chart of COVID-19 dashboard search process.

As part of the study's sampling procedure for the first round of data extraction, the dashboard's most recent year of data updates and/or the year of archival data were recorded. Any dashboard updates following the recorded date were not accounted for. In the first round, 25 dashboards were identified from the appendix of Barbazza et al. (27) and 23 from the Google search strategy. During the second round of data extraction, 38 dashboards were identified. After removing duplicates, a final total of 69 dashboards were included in the analysis. See Figure 1 for a flowchart illustrating the dashboard search process.

Across the manually coded subset, the average Cohen's kappa was 0.38, indicating fair agreement, consistent with the variability expected when coding heterogeneous, often loosely-defined dashboard features. Agreement was generally higher for structured, checkbox-based items (e.g., presence of specific indicators or visualization types) and lower for more interpretive fields requiring reviewer judgment, such as the stated target audience or purpose of the dashboard. Among the subset of variables central to this analysis (purpose, target audience, narrative explanation of dashboard functionality, presence of uncertainty information, type of uncertainty information, uncertainty display, and narrative explanation of uncertainty), the average kappa was 0.07, reflecting slight agreement (31). Notably, raw percent agreement remained high across nearly all variables regardless of kappa value, and any discrepancies identified during dual coding were resolved through the third-reviewer consensus process described above; kappa and raw agreement statistics for each variable are reported in Supplementary Table 1.

We defined the dashboard's geographic scope as the smallest geographic unit for which the data were aggregated. Dashboards created at the Ontario regional level were most common (22/69, 32%). The following provinces and territories had only one COVID-19 dashboard: Northwest Territories, Nunavut, Saskatchewan, and Prince Edward Island. It was also common to see more dashboards created in provinces with larger populations (e.g., Ontario, British Columbia, and Quebec).

Table 1 presents a summary of the key data extracted from the environmental scan of COVID-19 dashboards. Based on the data extraction criteria, the information is categorized under uncertainty information and metadata. For a comprehensive summary of the dashboard data, including the dashboard purpose, target audience, types of visualizations, types of public health indicators, technology platforms used, and functionality, please refer to Supplementary Table 2. A table containing the links to all analyzed dashboards is also included in Supplementary Table 3.

**Table 1:** Summary statistics from the extracted data.

Domain and Subdomains	N (%)
Uncertainty included	29 (42%)
Type of Uncertainty	
Geospatial	14 (20%)
Not Stated	10 (15%)
Statistical	5 (7%)
Type of Geospatial Uncertainty	
Temporal	13 (19%)
Spatial	3 (4%)
Attribute	2 (3%)
Other types of uncertainty	
Potential errors	2 (3%)
Technical Issues	1 (1%)
Changes in Case Definitions	1 (1%)
Display	
Text	29 (42%)
Numerical	1 (1%)
Visualization	1 (1%)
Narrative Explanation of Uncertainty	0 (0%)
Metadata	
Definitions of Public Health Indicators	37 (54%)
Model Assumptions	1 (100%)
Source of Dataset	47 (68%)
Narrative Explanation of Dashboard Functionality	33 (48%)
Downloadable Data	26 (38%)

Subcategories are non-mutually exclusive; totals exceed overall counts.

**Figure 2:**
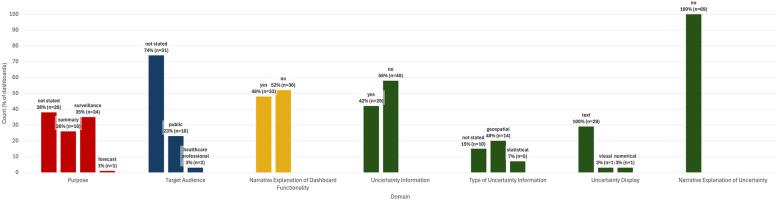
Grouped bar plot displaying primary findings.

Figure 2 is a grouped bar plot that illustrates the primary findings of the environmental scan, including summary statistics on the dashboard's purpose and target audience, a narrative explanation of its functional features, the presence of uncertainty information, the type of uncertainty information, the type of uncertainty display, and a narrative explanation of the uncertainty. Note that the bar plot displays mutually exclusive categories across all domains, but a dashboard can display multiple types of uncertainty. Therefore, the data for three dashboards were duplicated to include additional data that fit more than one category (e.g., an uncertainty display that included both numerical data and a visualization).

We discuss the main secondary findings from the data extraction. These findings include the types of public health indicators, types of visualizations, technology platforms, and types of interactive features found in the dashboards. The results are explained further in the following sections.

### 3.1 Creators, users, and uses of COVID-19 dashboards

Almost all COVID-19 dashboards (66/69, 96%) were created by publicly funded healthcare organizations, academic institutions, and government bodies. A few dashboards (3/69, 4%) were created by individual authors with no connections to publicly-funded institutions, and these were designated as “private” creators.

The majority of the dashboards (51/69, 74%) did not clearly state the target user or audience for the dashboard. When stated, the target user was often the general public (16/69, 23%). Only 2 of 69 dashboards (3%) were said to be created for use by healthcare (including public health) professionals.

The purpose of the dashboard was also not stated clearly in over a third of the dashboards (26/69, 38%). When stated, the purpose was often described as being for monitoring and surveillance of the community's health status (24/69, 35%). Eighteen of 69 dashboards (26%) were developed to act as a summary report of the community's COVID-19 data. Only 1 of 69 dashboards (1%) was designed for forecasting with predictive analytic features.

### 3.2 Content and data of COVID-19 dashboards

COVID-19 cases were used as a public health indicator in almost all dashboards (64/69, 93%). Hospitalizations (40/69, 58%), deaths (39/69, 57%), and risk measures (31/69, 45%) were also common indicators used. The complete list of indicators can be found in Supplementary Table 2 under Types of Indicators.

These public health indicators were represented in a variety of data visualizations, most commonly in a table (47/69, 68%). Other common visualizations and visual cues included a line graph (39/69, 57%), bar chart (32/69, 46%), and map (29/69, 42%).

### 3.3 Technology and functionality of COVID-19 dashboards

For most, it was not clear which technology platform was used to develop the dashboard. When this information was visible, it was found that most dashboards were developed using Microsoft's Power BI (11/69, 16%), Tableau (9/69, 13%), or Esri's ArcGIS (9/69, 13%).

The majority of the dashboards were interactive (46/69, 67%) with the most common interactive features being the sort/filter (19/46, 41%) and hover (18/46, 39%). Most dashboards, whether they were interactive or static, had filtered or grouped the data by region (29/69, 42%) and age (23/69, 33%).

### 3.4 Uncertainty and metadata in COVID-19 dashboards

**Figure 3:**
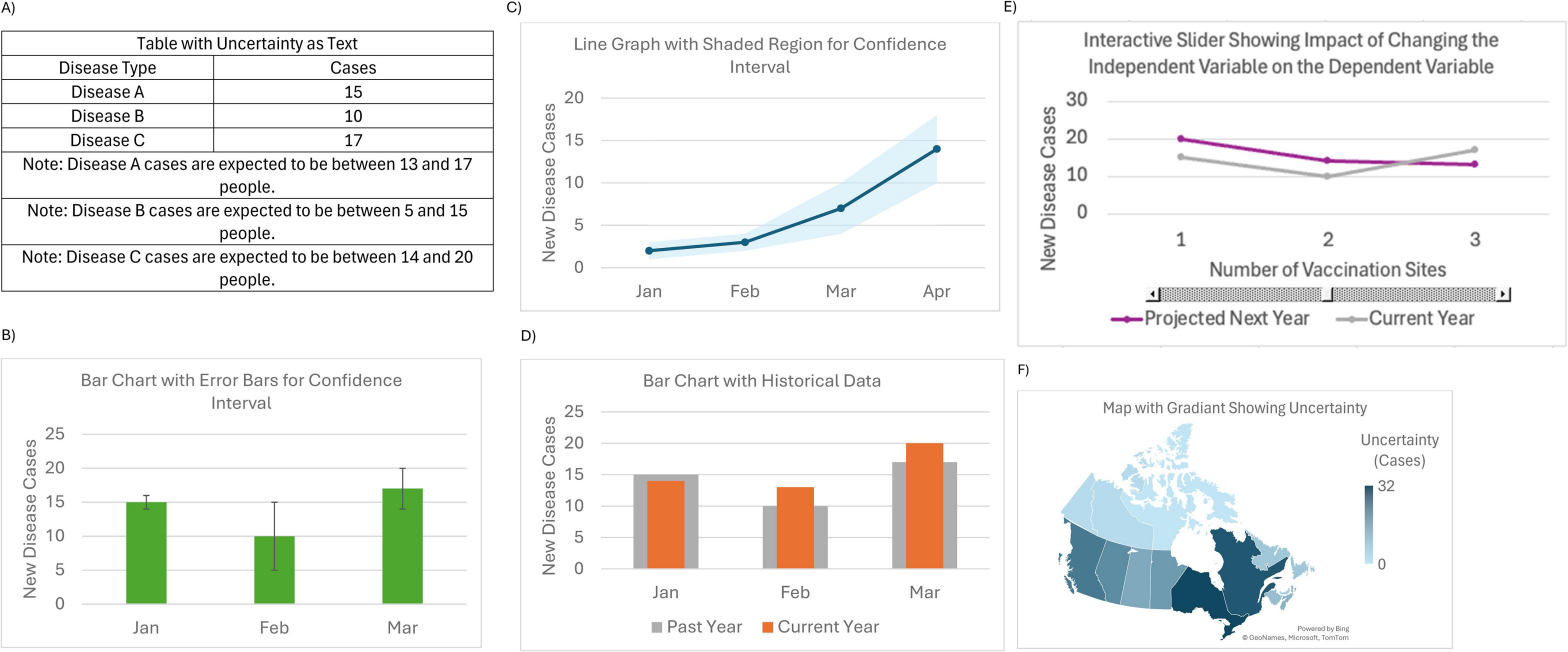
Examples of uncertainty displays: **(A)** table with uncertainty as a textual description, **(B)** bar chart with error bars containing the confidence interval, **(C)** line graph with shaded regions containing the confidence interval, **(D)** bar chart with overlapping historical data **(E)** line graph with interactive slider, and **(F)** map with gradient showing uncertainty. Map generated using Microsoft Excel (32); Microsoft product screenshot reprinted with permission from Microsoft Corporation.

Only 1 of 69 dashboards (1%) included a visualization of uncertainty (see Figure 3), and only 1 of 69 (1%) provided a numerical estimate of uncertainty. In contrast, 29 of 69 dashboards (42%) included some form of uncertainty information, and in nearly all cases, this was communicated exclusively through text. Among the dashboards that described uncertainty, most focused on geospatial sources, particularly temporal uncertainty related to reporting delays and data updates. However, even when uncertainty was acknowledged, it was rarely quantified or visualized, and 10 of the 29 dashboards did not specify the source of the uncertainty. Other types of uncertainty were identified that did not fit the predefined categories, including potential errors, technical issues, and changes in case definitions. None of the dashboards included a narrative explanation to support users' understanding of the uncertainty. These findings indicate that while uncertainty is sometimes acknowledged in pandemic dashboards, it is almost never operationalized through numerical or visual representations that would support interpretation or decision-making.

Most dashboards included definitions of the public health indicators/measures provided (37/69, 54%) and data sources (47/69, 68%). Only one dashboard used a forecasting model, and it included a description of the model assumptions.

## 4 Discussion

### 4.1 Principal findings

Despite extensive research on uncertainty visualization, our findings show that these approaches are almost entirely absent from Canadian COVID-19 dashboards. Only 1 of the 69 dashboards analyzed included a visualization of uncertainty, only 1 provided a numerical estimate, and all dashboards that acknowledged uncertainty relied almost exclusively on text-based descriptions. This gap is particularly concerning given the variability inherent in COVID-19 data, as it suggests the uncertainty data is not being reflected in how information is presented to users. Without explicit communication of uncertainty, users may interpret point estimates as definitive, potentially leading to overconfidence in the data and suboptimal public health decisions, such as misallocation of resources or misinterpretation of regional risk (11, 19). The absence of uncertainty visualizations in practice is a missed opportunity for transparency and a potential limitation affecting the efficacy of dashboards as decision-support tools. Identifying the factors contributing to this disconnect between research and practice is critical for improving the design and actionability of future public health dashboards.

In the few dashboards that provided uncertainty information, it was mostly displayed as text. This text typically described data limitations but did not provide guidance to support interpretation or decision-making. In one example, the authors state, “Please note: Every effort is made to keep this list up-to-date and complete, but it may not reflect all individuals tested. Please keep that in mind when reviewing this information.” This type of uncertainty statement acknowledges potential limitations in the data but does not support interpretation or decision-making. To make uncertainty information actionable, it must be quantified and accompanied by guidance that links uncertainty levels to the user's decision-making. This can be accomplished with a framework that models public health decision-making under uncertainty. If a framework for quantifying and communicating uncertainty is not established, public health risk forecasting and resource allocation decisions may become susceptible to inconsistent or biased decision-making.

### 4.2 Why are uncertainty visualizations absent?

The absence of uncertainty visualizations in Canadian COVID-19 dashboards raises important questions about the factors that may limit their adoption in practice. One potential explanation relates to assumptions about the target audience. Dashboard designers may prioritize simplicity and interpretability for a broad public audience and therefore, avoid incorporating uncertainty information due to concerns that it may increase cognitive burden or lead to confusion. This concern is consistent with longstanding challenges in risk communication, where presenting uncertainty may be perceived as undermining clarity or public confidence, which could explain the reliance on simplified dashboard designs observed in this study (4).

A second contributing factor may be technical and tooling constraints. Many dashboards in this study were developed using widely available platforms such as Power BI, Tableau, and ArcGIS, which may not readily support more advanced uncertainty visualizations such as hypothetical outcome plots, quantile dotplots, or ensemble displays. Implementing these techniques may require custom development or specialized expertise, which may not be feasible within the time and resource constraints under which many dashboards were developed.

A third explanation relates to the broader culture of risk communication in public health. The reliance on text-based disclaimers observed in this study suggests a tendency toward conservative communication strategies that emphasize clarity over nuanced representations of uncertainty, particularly during public health crises (4, 32, 33). In such contexts, uncertainty may be communicated in the form of brief textual disclaimers rather than integrated into the visual representation of the data. While this approach can preserve clarity or reduce the risk of immediate misinterpretation, it may limit users' ability to fully understand the variability and limitations of the data, potentially leading to miscalibrated trust or overconfidence in the information presented (4, 19, 23).

Finally, the findings suggest a broader disconnect between the uncertainty visualization research literature and real-world dashboard design practices. Over the past decade, researchers have developed and empirically evaluated a range of visualization techniques that improve users' ability to interpret uncertainty and make decisions under uncertainty. However, the lack of uptake of these approaches in public health dashboards indicates that these advances have not been widely translated into practice. This gap may reflect differences in priorities, communication goals, or collaboration between researchers, dashboard designers, and public health practitioners.

In summary, the absence of uncertainty visualizations may result from interacting constraints related to audience assumptions, technical feasibility, communication norms, and the translation of research into practice.

### 4.3 Implications for dashboard design and decision-making

The findings of this study highlight several implications for the design and use of public health dashboards, particularly in relation to how uncertainty is communicated and used in decision-making. Across the dashboards analyzed, the purpose and target users were often not clearly defined, and uncertainty information was rarely quantified, visualized, or accompanied by guidance on how it should inform decision-making. As a result, dashboards may present data without adequately supporting users in interpreting its limitations or applying it to real-world decisions.

A key implication is the need for clearer articulation of dashboard purpose. In the dashboards reviewed, purpose was often unclear, which may contribute to the lack of alignment between design and decision-making needs. When a purpose was stated, it was most commonly monitoring and surveillance. While this is appropriate in the context of a rapidly evolving public health crisis, it also implies specific user needs and decision contexts. For example, dashboards intended for surveillance may be used by public health professionals to track trends and allocate resources, whereas dashboards intended for public communication may prioritize accessibility and risk interpretation. Without a clearly defined purpose, it becomes difficult to design appropriate visualizations, including how uncertainty should be represented and communicated.

Closely related to purpose is the identification of target users. The absence of clearly defined target users observed in this study suggests that dashboards may not be designed to support the needs of specific user groups. Potential users of public health dashboards range from highly trained professionals, such as epidemiologists, to members of the general public with varying levels of statistical and domain knowledge. These groups differ substantially in their ability to interpret complex data and uncertainty information. Designing dashboards without considering these differences may limit their effectiveness, as visualizations that are appropriate for expert users may be inaccessible to non-experts, while overly simplified displays may not support expert decision-making.

A particularly important implication relates to how uncertainty is communicated. Nearly all dashboards that included uncertainty relied exclusively on text-based descriptions, typically in the form of general statements about data limitations, reporting delays, or completeness. The reliance on text-based uncertainty descriptions observed in this study may limit users' ability to interpret the magnitude of uncertainty, as these formats provide less precise guidance than numerical or visual representations (35–37). In this context, text-based uncertainty communication may function more as a disclaimer than as a decision-support mechanism.

These findings can also be interpreted through the lens of Han et al.'s taxonomy. most uncertainty described in the dashboards(e.g., reporting delays, testing limitations, and changes in case definitions) corresponds to ambiguity, arising from incomplete or imperfect information (3). However, this type of uncertainty was typically communicated through brief textual statements without quantification or structured representation. In contrast, probabilistic uncertainty, which could be expressed through numerical ranges or visual distributions, was almost entirely absent. This mismatch suggests that dashboards may not be aligning their communication strategies with the underlying sources of uncertainty, limiting users' ability to interpret and act on the information provided. Applying frameworks such as Han et al.'s could support the development of more targeted and actionable approaches to uncertainty communication in public health dashboards.

These findings highlight the need for user-centred design approaches that explicitly account for differences in expertise and decision-making needs. One potential strategy is to incorporate layered or adaptive interfaces that allow users to engage with information at different levels of complexity, including optional access to more detailed uncertainty information. Additionally, dashboards should provide clear narrative explanations or guidance to support interpretation, particularly when uncertainty is involved. Although many dashboards included definitions of public health indicators, few provided explanations of how to interpret the data or its associated uncertainty. This lack of guidance may limit the usability of dashboards, especially for non-expert users, and reduce their effectiveness as decision-support tools.

Improving the actionability of public health dashboards requires the inclusion of uncertainty information and careful consideration of dashboard purpose, target users, and the provision of interpretive guidance. Aligning these design elements can help ensure that uncertainty information is meaningfully integrated into decision-making processes.

### 4.4 Strengths and limitations

This study builds on existing literature regarding the features of actionable public health dashboards and provides, to our knowledge, the most comprehensive review of Canadian COVID-19 dashboards to date. By combining manual data extraction with AI-assisted methods, the study was able to capture a broader set of dashboards than would have been feasible using traditional search strategies alone. In addition, this is one of the first studies to systematically examine how uncertainty information is communicated in public health dashboards, offering novel insights into an important but underexplored aspect of dashboard design.

Several limitations should be considered when interpreting these findings. First, the environmental scan was limited to publicly available dashboards, and it is possible that internal dashboards used within public health organizations were not captured. These internal tools may differ in their use of uncertainty information and may be designed for more specialized audiences.

Second, although efforts were made to ensure consistency in data extraction through reviewer training and independent verification, some categories, particularly those involving interpretation of textual descriptions (e.g., dashboard purpose, target audience, and uncertainty explanations), remain subject to reviewer interpretation. While discrepancies were resolved through consensus, this introduces the possibility of residual subjectivity in the dataset.

Third, a substantial portion of the dashboard sample was identified and processed using AI-assisted tools (ChatGPT with the Scraper and Make a Sheet plugins), which introduces additional methodological considerations. The workflow relied on a consumer-facing chat interface and third-party plugins that have since been deprecated, limiting the reproducibility of the exact extraction process. In addition, because multiple dashboards may have been processed within the same session, there is a possibility of context carryover, where earlier outputs influenced subsequent extractions. Furthermore, many dashboards in the sample were built using JavaScript-based platforms (e.g., Power BI, Tableau, ArcGIS), and it is unclear whether the scraping tools fully rendered dynamic content. If only partial or static HTML representations were captured, some dashboard elements, particularly interactive or visually embedded uncertainty displays, may have been missed, potentially leading to underestimation of uncertainty visualization usage.

To mitigate these risks, all AI-extracted data were manually reviewed and verified by multiple reviewers, and corrections were made where necessary. However, these limitations highlight the need for more transparent and reproducible workflows when incorporating AI tools into research processes.

Finally, the study did not capture certain contextual factors that may influence dashboard usability and interpretation, such as language accessibility, user interface design quality, or user engagement patterns, and it did not include user testing to evaluate how real users interpret and act on dashboard information. In addition, because this study focused exclusively on Canadian COVID-19 dashboards, the findings may not be fully generalizable to dashboards developed in other countries with different public health systems, data infrastructures, and design practices. Future work could extend this analysis by incorporating user-centred evaluations and usability testing to better understand how different audiences interpret and use dashboard information, including uncertainty displays.

### 4.5 Future research

Future research should build on the conceptual and practical gaps in public health dashboard design and decision-making identified in this study. A key priority is developing a clearer understanding of the purpose and use cases of public health dashboards. While this study adopted a taxonomy based on functionality and interactivity, additional frameworks may be needed to better capture the range of decision-making contexts in which dashboards are used, including public communication, surveillance, and policy planning. Clarifying these use cases would provide a stronger foundation for aligning dashboard design with intended outcomes.

Closely related is the need to better define and account for target users. Public health dashboards are often publicly accessible but may serve audiences with widely varying levels of statistical and domain expertise, from epidemiologists to the general public. Future work should explore how dashboards can be designed to accommodate this variability, for example through user-centred design approaches, layered interfaces, or adaptive features that allow users to engage with information at different levels of complexity. Particular attention should be given to how uncertainty information is presented, ensuring that it is both interpretable and actionable for different user groups, including public health professionals with varying levels of statistical training. For example, recent work has begun to investigate user-centred approaches to uncertainty communication in this context (38).

A more detailed qualitative analysis of how uncertainty is communicated in dashboards represents another important avenue for future research. For example, uncertainty statements could be categorized as disclaimers, limitations, or actionable guidance to better understand their role in supporting (or limiting) decision-making. However, because some dashboards are no longer accessible or have changed since data collection, a complete and systematic analysis of the original text is no longer feasible. Future work could address this limitation by capturing and archiving dashboard content to enable more rigorous qualitative coding and longitudinal analysis.

There is also a need for greater transparency and formalization of public health decision-making processes. Resource allocation and policy decisions during a public health crisis are influenced by empirical data and contextual factors such as local policies, precautionary principles, stakeholder interests, and institutional constraints (39). Developing clearer models of how these factors interact with data, including uncertainty, would provide valuable guidance for the design of decision-support tools. Such work could also inform the development of best practices and training frameworks to support the effective use of dashboards in public health contexts.

Finally, future research should investigate how uncertainty visualizations can be effectively designed for public health applications. Advances in visualization research have produced a range of techniques for communicating uncertainty, yet these have not been widely adopted in practice. Understanding how to adapt these approaches to geospatial and epidemiological data, while maintaining usability and reducing cognitive load, remains an open challenge. Interactive and user-centred visualization strategies may be particularly promising, as they allow users to explore uncertainty dynamically and tailor the interface to their needs. Addressing these questions will be critical for developing dashboards that actively support informed and robust decision-making under uncertainty.

## 5 Conclusion

This study increases knowledge about the creators of Canadian COVID-19 dashboards and sources for public health data. This information can be leveraged for collaborations and the development of tools that link the data and resources from external organizations as the input in future public health dashboards. This research also highlights the target audience of Canadian COVID-19 dashboards and the implications of creating dashboards that will become publicly available. Furthermore, making the dashboard purpose clear can be an area of future research that leads to the development of a conceptual framework to categorize public health dashboard use cases. This information can support the design of user-centred narrative explanations, interactive features, and visualizations. Uncertainty information is underutilized in public health dashboards. With the future increase of predictive decision-support tools, more research is needed regarding how public health professionals make decisions using uncertainty information and how uncertainty information can be displayed in user-centred visualizations.

## Data Availability

The original contributions presented in the study are included in the article/Supplementary Material, further inquiries can be directed to the corresponding author/s.
