# Supplementary material for: Uncertainty displays in pandemic dashboards: a review of COVID-19 use cases
Source: Front Digit Health. 2026 Jul 30;8:1869108. doi: 10.3389/fdgth.2026.1869108 (PMC13469246; doi:10.3389/fdgth.2026.1869108)
Supplement: Supplementary file 1 [file Table1.docx]

Supplementary Material

# 1 AI Prompts for Dashboard Retrieval and Data Extraction

*Retrieval* (Scraper plugin)

Using the Scraper plugin, retrieve the visible text and page content from this dashboard URL: [URL]. Include titles, axis and chart labels, captions, footnotes, indicator definitions, and any notes on data sources, updates, or limitations.

*Standardized extraction*

From the retrieved content, extract the following fields for this Canadian COVID-19 dashboard. If a field is not stated, record "Not stated."

- Creator type (public / private)
- Purpose (monitoring/surveillance, summary report, forecasting)
- Target audience (public, health professionals)
- Visualization types (table, line graph, bar chart, map, pie chart, colour-coding, symbols)
- Public health indicators (cases, hospitalizations, deaths, recoveries, risk measures, vaccination, variants, population)
- Technology / platform, if identifiable
- Functionality (interactive / static) and interactive features (sort/filter, hover, show/hide, highlight, zoom)
- Filters (region, age, date, sex, indicator)
- Uncertainty: present or not; type (geospatial, statistical); geospatial subtype (temporal, spatial, attribute); display format (text, numerical, visualization); narrative explanation present or not
- Metadata: indicator definitions, forecasting model assumptions, data sources, narrative explanation of functionality, downloadable data

*Formatting* (Make A Sheet plugin)

Using the Make A Sheet plugin, organize the extracted fields into one table row, one column per field above, for compilation across dashboards.

# 2 Interrater Reliability

**Table 1.** Cohen’s Kappa for qualitative dashboard coding

| **Variable** | **Dashboards Compared (n)** | **Dashboards Excluded** | **Excluded Dashboard IDs** | **Raw % Agreement** | **Cohen's Kappa** | **Agreement Level** | **Note** |
| --- | --- | --- | --- | --- | --- | --- | --- |
| *Purpose* | 29 | 4 | 11, 30, 33, 40 | 34.5% | 0.086 | Slight |  |
| *Target Audience* | 31 | 2 | 9, 30 | 45.2% | -0.033 | Poor |  |
| *Narrative Explanation of Dashboard Functionality* | 33 | 0 |  | 48.5% | 0.06 | Slight |  |
| *Presence of Uncertainty Information* | 33 | 0 |  | 66.7% | 0.195 | Slight |  |
| *Type of Uncertainty Information* | 33 | 0 |  | 86.4% | 0.02 | Slight |  |
| *Uncertainty Display* | 33 | 0 |  | 88.9% | 0.12 | Slight |  |
| *Narrative Explanation of Uncertainty* | 4 | 29 | 1, 2, 3, 4, 5, 6, 7, 8, 9, 11, 12, 13, 15, 16, 18, 20, 22, 23, 28, 30, 31, 33, 34, 35, 37, 38, 39, 40, 41 | 100.0% | Undefined | N/A (undefined) | Undefined (perfect agreement, no variability) |
| AVERAGE | **κ: n=7 vars** |  |  | **67.2%** | **0.074666667** |  |  |

Cohen's kappa calculated using standard formula: κ = (P_o − P_e) / (1 − P_e)

Note: n = 33 dashboards included in kappa calculations. A further 6 independently coded dashboards were excluded as pre-consensus independent ratings were not retrievable for those reviews; only post-consensus scores were available.

# 3 Supplementary Dashboard Data

Table 2. Summary statistics from the extracted data

| Domain and Subdomains | N (%) |
| --- | --- |
| **Creator** |  |
| Public | 66 (96%) |
| Private | 3 (4%) |
| **Description** |  |
| *Purpose* |  |
| Not Stated | 26 (38%) |
| Monitoring/ Surveillance | 24 (35%) |
| Summary Report | 18 (26%) |
| Forecasting | 1 (1%) |
| *Audience* |  |
| Not Stated | 51 (74%) |
| Public | 16 (23%) |
| Health Professionals | 2 (3%) |
| *Type of Visualizations and Other Visual Cues* |  |
| Table | 47 (68%) |
| Line graph | 39 (57%) |
| Bar chart | 32 (46%) |
| Map | 29 (42%) |
| Colour-coding | 26 (38%) |
| Pie chart | 10 (14%) |
| Symbols (ex. arrows) | 9 (13%) |
| Other | 3 (4%) |
| *Types of Indicators* |  |
| Cases (before or after vaccination) | 64 (93%) |
| Hospitalizations | 40 (58%) |
| Deaths | 39 (57%) |
| Recovered (from COVID) | 32 (46%) |
| Risk Measures (ex. people testing, rate of transmission) | 31 (45%) |
| Other | 19 (28%) |
| Vaccination Rates | 13 (19%) |
| Variants (ex. omnicron, delta) | 11 (16%) |
| Population Size | 4 (6%) |
| *Technology Used* |  |
| Not Specified | 38 (55%) |
| Microsoft (Power BI) | 11 (16%) |
| Tableau | 9 (13%) |
| Esri (ArcGIS) | 9 (13%) |
| RShiny | 1 (1%) |
| Java Script (Charts & Maps) | 1 (1%) |
| **Functionality** |  |
| Interactive | 46 (67%) |
| Static | 23 (33%) |
| *Interactive Features* |  |
| Sort/ Filter | 19 (28%) |
| Hover (i.e. information pops up as you move your mouse) | 18 (26%) |
| Show/ Hide Information | 12 (17%) |
| Highlight (i.e. visual emphasis after clicking) | 9 (13%) |
| Zoom into Map | 2 (3%) |
| Expand and Condense Graph | 2 (3%) |
| *Filters* |  |
| Region (i.e. province, health unit) | 29 (42%) |
| Age | 23 (33%) |
| Date (ex. year, month) | 16 (23%) |
| Sex | 15 (22%) |
| Public Health Indicator | 12 (17%) |
| Vaccination information | 2 (3%) |
| Ethnicity | 1 (1%) |
| Confirmed vs. Probable Data | 1 (1%) |
| Role (i.e. patient vs. healthcare staff) | 1 (1%) |
| **Uncertainty** | **29 (42%)** |
| *Type of Uncertainty* |  |
| Geospatial | 14 (20%) |
| Not Stated | 10 (15%) |
| Statistical | 5 (7%) |
| *Type of Geospatial Uncertainty* |  |
| Temporal | 13 (19%) |
| Spatial | 3 (4%) |
| Attribute | 2 (3%) |
| *Other Types of Uncertainty* |  |
| Potential Errors | 2 (3%) |
| Technical Issues | 1 (1%) |
| Changes in Case Definitions | 1 (1%) |
| *Display* |  |
| Text | 29 (42%) |
| Numerical | 1 (1%) |
| Visualization | 1 (1%) |
| Narrative Explanation of Uncertainty | 0 (0%) |
| **Metadata** |  |
| Definitions of Indicators | 37 (54%) |
| Model Assumptions* | 1 (100%) |
| Source of Dataset | 47 (68%) |
| Narrative Explanation of Dashboard Functionality | 33 (48%) |
| Downloadable Data | 26 (38%) |

*Note that model assumptions would only be required for a forecasting dashboard, of which there was only one.

**Table 3.** Dashboard sources

|  | Dashboard Link |
| --- | --- |
| 1 | <https://archive.vn/ujVQi> |
| 2 | <https://archive.vn/DW6Ow> |
| 3 | <https://art-bd.shinyapps.io/covid19canada/> |
| 4 | <https://archive.vn/p7Ix2> |
| 5 | <https://archive.vn/Lqk6L> |
| 6 | <https://archive.vn/hQ9Kl> |
| 7 | <https://archive.vn/Tspb3> |
| 8 | <https://archive.vn/lT4SS> |
| 9 | <https://archive.vn/Dfwbz> |
| 10 | [https://archive.vn/xEOZl#cases](https://archive.vn/xEOZl#cases%20) |
| 11 | <http://archive.vn/xv4sw> |
| 12 | <http://archive.vn/UNPBb> |
| 13 | <http://archive.vn/LOfCa> |
| 14 | <http://archive.vn/HLobA> |
| 15 | <http://archive.vn/7T6MT> |
| 16 | <http://archive.vn/YtJVv> |
| 17 | <http://archive.vn/1Urge> |
| 18 | <http://archive.vn/3ZL3k> |
| 19 | <http://archive.vn/oXtDj> |
| 20 | <http://archive.vn/jky19> |
| 21 | [http://archive.vn/D3JfB on 14.07.20http://archive.vn/D3JfB](http://archive.vn/D3JfB%20on%2014.07.20) |
| 22 | <http://archive.vn/kVUii> |
| 23 | <http://archive.vn/NoyJh> |
| 24 | <http://archive.vn/jPIlp> |
| 25 | <http://archive.vn/cG5a0> |
| 26 | <http://www.bccdc.ca/health-professionals/data-reports/covid-19-surveillance-dashboard> |
| 27 | <https://files.ontario.ca/moh-covid-19-report-en-2020-06-12.pdf> |
| 28 | <https://www.publichealthontario.ca/en/data-and-analysis/infectious-disease/covid-19-data-surveillance/covid-19-data-tool?tab=overview> |
| 29 | <https://www.publichealthontario.ca/-/media/documents/ncov/epi/2020/06/covid-19-epi-diversity.pdf?la=en> |
| 30 | <https://www.toronto.ca/home/covid-19/covid-19-pandemic-data/> |
| 31 | <https://www.toronto.ca/home/covid-19/covid-19-pandemic-data/covid-19-archived-dashboards/> |
| 32 | <https://art-bd.shinyapps.io/covid19canada/> |
| 33 | <https://resources-covid19canada.hub.arcgis.com/> |
| 34 | <https://www.bankofcanada.ca/markets/market-operations-liquidity-provision/covid-19-actions-support-economy-financial-system/covid-19-stringency-index/> |
| 35 | <https://covid19-sciencetable.ca/ontario-dashboard/> |
| 36 | <https://ourworldindata.org/explorers/coronavirus-data-explorer> |
| 37 | <https://www.ottawapublichealth.ca/en/reports-research-and-statistics/covid19-dashboard.aspx> |
| 38 | <https://health-infobase.canada.ca/covid-19/current-situation.html> |
| 39 | <https://health-infobase.canada.ca/covid-19/#a8> |
| 40 | <https://dashboard.clsa-elcv.ca/clsa-dashboard-en/> |
| 41 | <https://www150.statcan.gc.ca/n1/en/subjects/society_and_community/coronavirus?conttype_and=2024#data> |

*Note: Some links contain multiple dashboard pages.
